# Visual steady state in relation to age and cognitive function

**DOI:** 10.1371/journal.pone.0171859

**Published:** 2017-02-28

**Authors:** Anna Horwitz, Mia Dyhr Thomsen, Iris Wiegand, Henrik Horwitz, Marc Klemp, Miki Nikolic, Lene Rask, Martin Lauritzen, Krisztina Benedek

**Affiliations:** 1 Department of Neuroscience and Pharmacology, University of Copenhagen, Blegdamsvej 3, Copenhagen, Denmark; 2 Center for Healthy Aging, University of Copenhagen, Blegdamsvej 3, Copenhagen, Denmark; 3 Department of Clinical Neurophysiology, Rigshospitalet–Glostrup, Nordre Ringvej 57, Glostrup, Denmark; 4 Department of Psychology, University of Copenhagen, Øster Farimagsgade 2A, Copenhagen, Denmark; 5 Department of Clinical Pharmacology, Bispebjerg Hospital, Bispebjerg Bakke 23, København NV, Denmark; 6 Department of Economics and Population Studies & Training Center, Brown University, Providence, Rhode Island, United States of America; 7 Department of Economics, University of Copenhagen, Øster Farimagsgade 5, Copenhagen, Denmark; University of Electronic Science and Technology of China, CHINA

## Abstract

Neocortical gamma activity is crucial for sensory perception and cognition. This study examines the value of using non-task stimulation-induced EEG oscillations to predict cognitive status in a birth cohort of healthy Danish males (Metropolit) with varying cognitive ability. In particular, we examine the steady-state VEP power response (SSVEP-PR) in the alpha (8Hz) and gamma (36Hz) bands in 54 males (avg. age: 62.0 years) and compare these with 10 young healthy participants (avg. age 27.6 years). Furthermore, we correlate the individual alpha-to-gamma difference in relative visual-area power (Δ*R*_*V*_) with cognitive scores for the older adults. We find that Δ*R*_*V*_ decrease with age by just over one standard deviation when comparing young with old participants (*p*<0.01). Furthermore, intelligence is significantly negatively correlated with Δ*R*_*V*_ in the older adult cohort, even when processing speed, global cognition, executive function, memory, and education (*p<*0.05). In our preferred specification, an increase in Δ*R*_*V*_ of one standard deviation is associated with a reduction in intelligence of 48% of a standard deviation (*p<*0.01). Finally, we conclude that the difference in cerebral rhythmic activity between the alpha and gamma bands is associated with age and cognitive status, and that Δ*R*_*V*_ therefore provide a non-subjective clinical tool with which to examine cognitive status in old age.

## 1 Introduction

### 1.1 Overview

The relationships between steady-state VEP power-response (SSVEP-PR) and higher cognitive function are not fully understood [1–3]. However, researchers have found that neurophysiological changes tend to precede cognitive decline, and that subjects with dementia show electrophysiological changes, suggesting that EEG-related changes may usefully predict cognitive function and decline [4–7]. In this study, we show that a novel non-task stimulation-induced EEG oscillations can robustly predict cognitive status in a birth cohort of healthy Danish males (Metropolit) with varying cognitive ability. This article presents the first set of results based on measurements of the present novel stimulation design using the Metropolit cohort.

The visual evoked potential (VEP) is the EEG response to a visual stimulus, and has proven to be a convenient method with which to investigate visual and cognitive processing [1–3;8]. Steady-state VEP responses (SSVEP) are evoked by stimuli that, through their constant high frequency presentation, prevent neural activity from recovering baseline values before the next stimulus. The result is a continuous sinusoidal response [9] that is a function of the number of pyramidal cells firing in synchrony with the visual flicker and varies as a function of the temporal frequency of the driving stimulus [10;11]. SSVEP-PRs can be provoked by flickering an image at a frequency above 3 Hz [12]. Importantly, SSVEPs demonstrate more robust, higher signal-to-noise, and lower artifact ratios than the transient VEPs that are evoked at lower stimulation frequencies. Therefore, the SSVEP-PR may prove to be a useful phenomenon with which to generate robust quantitative clinical data.

We constructed a novel measure of the change in the brain’s relative visual-area power response between stimulation at low and high flicker rates and investigated two related issues. First, we investigated how the measure varies across young and old participants. Second, we investigated the association between the measure and cognitive function in the old participants. We find that the measure is significantly correlated with both age and cognitive function. Furthermore, we show that alternative candidate measures for the frontal and the parietal areas do not have similar explanatory power.

A central element of the analysis is the variation in the flicker rate of the visual stimulus. Varying the flicker rate enable us to study the SSVEP-PR in both the gamma and alpha bands, and, importantly, to study the difference between the power responses in these two frequency bands. Activity in the gamma band is associated with Alzheimer’s disease, which prompted our interest in recording individual gamma band activity in relation to cognitive function. Since the SSVEP-PR may vary between individuals along with confounding unobserved variables we focus on a normalized measure, namely the alpha-to-gamma difference in the ratio of power in the visual-area to a reference area. Given the prominence of the visual system in the brain, as well as the visual nature of our stimulation procedure we focus on the visual area.

Our main explanatory variable is therefore the difference in this relative visual-area power between the alpha and gamma frequencies as a consequence of a visual stimulus flickering at either 8 or 36 Hz as well as investigated for the frontal area.

The existing literature has found that there are long-range connections between the parietal–occipital and the frontal regions and that these areas are main sources for SSVEP responses [13–14]. For this reason, we also investigated the frontal region using a similar measure.

We present and test the hypothesis that the alpha-to-gamma relative visual-area power response reflects a key element of cognitive function. In particular, we establish that this novel measure is both related to age (which is itself related to cognitive function) and intelligence, indicating that indeed the alpha-to-gamma relative visual-area power response reflects a key element of cognitive function.

### 1.2 Context and focus

Previous studies have provided mixed evidence for a link between EEG measurements and cognitive function. For example, Wilson and O’Donnell investigated the relationship between SSVEP latency and mental workload without finding any reliable associations [15]. Conversely, some later studies of younger adults have revealed that the SSVEP amplitude is reduced during encoding of working memory [3;8]. Furthermore, it has been found that endogenous attention modulates the amplitude the magnitude of neural population activity, and phase coherence, reflect neural response synchronization, of SSVEPs [16;17]. Attentional modulation of SSVEP amplitude may reflect a control mechanism for neural response gain that selectively enhances attended signals in early visual cortical areas [18–23]. Meanwhile, research on EEG measurements of individuals suffering from Alzheimer’s disease has uncovered various associations between Alzheimer’s disease and EEG measurements in different frequency spectra. In particular, researchers have found that Alzheimer’s disease patients exhibit higher EEG power in the delta and theta bands, and lower power in the alpha and beta bands [4–6;24–25]. In addition, researchers have found increased 40 Hz auditory steady-state responses in Alzheimer’s disease patients with mild to moderate cognitive decline [24].

The observed associations between EEG measurements and Alzheimer’s disease suggest the possibility of a general link between cognitive function (e.g., intelligence) and EEG power. In particular, EEG measurements may reflect variations in cognitive function across individuals. To our knowledge, this present study presents the first direct evidence of a link between intelligence and SSVEP measurements, using a healthy group of age and gender-wise homogenous subjects. We present a novel EEG measure of within-individual SSVEP power differences in response to visual stimulation at two different stimulation frequencies and find that this measure statistically significantly predicts cognitive function of our study subjects.

A central question in the field of cognitive aging is whether age-related decline in intelligence is caused by a decline in processing speed or frontal-executive function [26–27]. The present study contributes to this discussion by controlling statistically for a range of factors capturing processing speed (i.e., the Trail Making A score) and executive function (i.e., the Trail Making B score and the Symbol Digit Modalities Test score). Furthermore, we control for variation in the individual’s memory (i.e., the Paired Associative Learning test score), global cognition (i.e., the Addenbrooke’s Cognitive Examination score), as well as an important socioeconomic factor (i.e., their years of education). Interestingly, we find that the association between our EEG measure and intelligence is robust to controlling for all of these other factors. This suggests that our EEG measure reflects a constituent component of intelligence that is not related to these factors.

Some existing studies have pointed to a significant age-dependence and have reported a decrease in power in the gamma-band with age, although little is known about gamma responses over the lifetime of subjects [28–33]. An early study investigating alpha frequency responses find that although it diminished with age, the amplitude did not demonstrate a high age correlation [34]. Here, we also want to address the age-dependent association and we therefore compared the EEG measures in a group of young adults with the measures obtained in our main cohort of older, age-matched males. We find that our EEG measure is correlated with age of subjects, suggesting that its association with intelligence also capture age-related cognitive factors.

We chose to focus on alpha-band and gamma-band related EEG responses for a variety of reasons. Gamma-band responses have been associated with higher-order cognitive functions and are considered a key to understanding neural signature information processing [35–39]. Furthermore, studies investigating attention and SSVEP find that the SSVEP alpha band power (8 and 12 Hz) increased at electrodes over both the occipital and the parietal cortex when subjects’ increased their attention [21;40].

Furthermore, we chose to focus on the visual region (relative to a reference region) due to the visual nature of the stimulation. Furthermore, of the sensory systems, the visual system has largest amount of neurons in the brain dedicated to processing sensory information. Therefore, we expect that the EEG power in the occipital region to carry more relevant information than that of other regions.

Overall, we show that the EEG response to a simple non-task visual stimulation in the gamma and alpha range in a cohort of healthy males is correlated with intelligence. The perspectives are wide: we believe that our results suggest that the present methodology could be used as a non-invasive, inexpensive, simple, and easily-used non-subjective clinical tool with which to examine cognitive status.

## 2 Materials and methods

EEGs were recorded while participants were presented with a flickering complex image at either a low or a high flicker rate to evoke SSVEP-PRs in the alpha and gamma ranges.

We initially investigated the effect of age on SSVEP-PR in response to stimulation with a flickering image (see Section 2.2). We assessed two cohorts, the first being an age-matched, older male cohort, and the second, a group of young adults of either gender. We subsequently investigated the correlation between SSVEP-PR and cognitive function in the older, all-male, age-matched cohort (see Section 2.1) [41–42].

The present research contributes to the field by attempting to tackle a key statistical issue related to heterogeneity between individuals in unobserved factors that may affect both EEG measurements and intelligence. In particular, one might worry that the level of EEG responses to visual stimulation may reflect not only the outcome measure of interest (in this case, intelligence), but also, e.g., individual-specific or study-day-specific factors that are related to subjects in clinically uninteresting ways. For example, it is possible that personal stress levels of individuals affect not only their performance on the intelligence test but also their EEG measures. We therefore attempt to normalize our variable of interest in two dimensions. Firstly, we focus on EEG power in the visual scalp region relative to that of a reference region (the main results are robust to the use of alternative reference regions, e.g., the whole scalp). Secondly, we focus on the difference in this measure in response to stimulation at the alpha and the gamma frequency. These normalizations are designed to account for individual-specific constant factors not affecting power relatively more or less in the occipital region or individual-specific constant factors not affecting power in the alpha and gamma ranges differentially. The precise definition of the variable is described in Section 2.8 below. We argue that this doubly normalized measure largely accounts for individual-fixed heterogeneity in unobserved factors that may affect observed intelligence.

Furthermore, as mentioned above, the present study also controls statistically for a range of factors capturing potential mechanisms that may generally be related to cognitive decline (i.e., the Trail Making A score, the Trail Making B score, the Symbol Digit Modalities Test score, the Paired Associative Learning test score, the Addenbrooke’s Cognitive Examination score, and the years of education).

### 2.1 Subjects

#### 2.1.1 Cohort 1: 54 age-matched elderly men

The first cohort was an age- and gender-matched group of similar (homogeneous) elderly participants drawn from the Metropolit 1953 Danish Male Birth Cohort (Metropolit) [41–42]. Our sample comprised 54 men born in the Danish capital region of Copenhagen in 1953 (meaning that they were aged 61 or 62 at the time of investigation). The average age was 61.9 years. Eighty-six percent of the subjects were right-handed, with normal or corrected-to-normal vision. The subjects had neither neurologic nor psychiatric disorders. Furthermore, all participants had structural MR scans carried out, none of which revealed any evidence of significant abnormalities.

The test-persons in our sample were additionally examined with neurocognitive testing. The average Mini-Mental State Examination (MMSE) score was 29.40 (range: 27–30). Furthermore, the average Addenbrookes Cognitive Examination (ACE) score was 93.83 (range: 82 to 100; S1 Table).

#### 2.1.2 Cohort 2: Ten young adults

The second sample comprised 10 healthy subjects with a mean age of 27.6 years (range: from 25 to 34 years). Five subjects were male and five were female. Nine of the subjects were right-handed, and one, left-handed; all 10 had normal or corrected-to-normal vision. The subjects had neither neurological nor psychiatric disorders, and all provided written informed consent.

### 2.2 Visual stimuli

The stimulation design involved showing the participants a flickering image (a black/white ‘Rubin’s vase’ [43]) in a non-task sensory steady-state stimulation.

**Fig 1:**
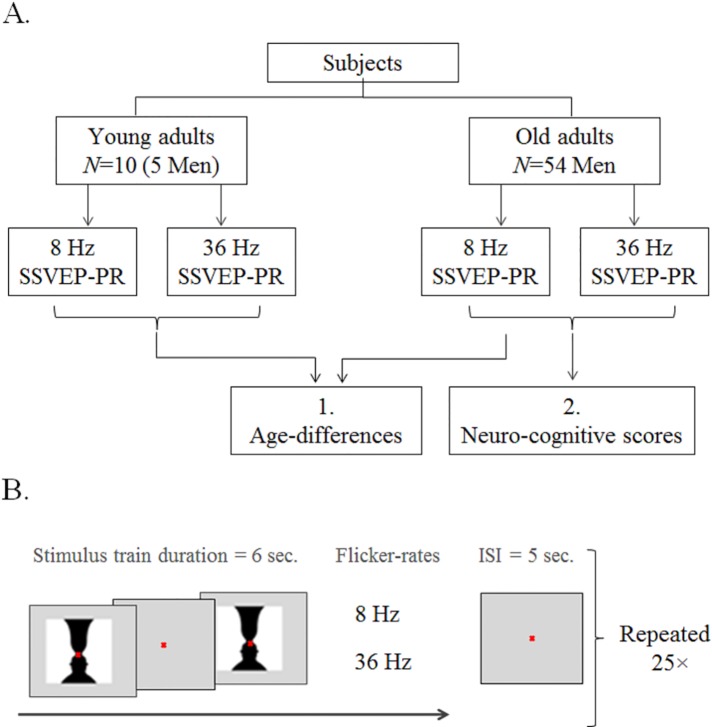
Study design. Panel A) Flowchart of the subject groups and stimulations. Box 1 illustrates the sample used to investigate the effect of age and flicker-rate on steady-state VEP power responses evoked by a complex image. Box 2 illustrates the sample used to investigate the correlation between intelligence and steady-state VEP measurements. Panel B) Schematic illustration of the stimulation procedure, with 6-second stimulation epochs, each indicated by a trigger. The abbreviation “ISI” denotes Inter-Stimulus Interval.

Each stimulus consisted of an “on/off design” where the image was shown flickering for 6 seconds. In general, the strongest SSVEP-PR was obtained by stimulation frequencies at 10, 20, or 40 Hz [10]. We choose the high flicker rate to be 36 Hz, i.e., in the gamma range, which is as near as possible to the 40 Hz frequency. We chose the low flicker rate to be at 8 Hz, i.e., in the low alpha range [12;44]. 5-second intervals separated each stimulus train of 6 seconds. These blocks of stimuli, with intervals, were repeated 25 times (Fig 1), resulting in a total of 1600 person-epochs or 102.400 electrode-epochs. All subjects were presented both simulations and were able to see the flickering image at both frequencies.

The size of the image was designed to span the central visual area, and measured 5 degrees horizontally and 3.25 degrees vertically (size: 8.72 cm × 5.72 cm). For the duration of the stimulation, a red fixation cross was superposed (size: 0.33 mm) at the middle of the screen.

### 2.3 Data acquisition

A soft elastic cap with 64 surface electrodes (Ag/AgCl) was used, with electrodes placed according to the international 10–20 system. A CURRY 7.4 Neuroscan was used for signal acquisition and data processing. The ground electrode was placed over the midline frontal region, and the reference (not to be confused with the reference area electrodes, as defined in Section 2.8), between the Cz and Pz-electrodes. For data analysis, the signals were re-referenced to bimastoid electrodes (M1, M2). We chose to reference the signal to the mastoid electrodes out of concern of clinical relevance.

Electrode impedance was maintained below 5 kΩ. Four additional electrodes were placed above, below, and lateral to the eyes, to correct for off-line electro-oculographic artifacts. Two EKG-electrodes were placed at the heart axis, and two electrodes were placed submentalis to record EMG-artifacts. EEG amplitudes were sampled at 2 kHz with an analog, first-order, anti-aliasing RC low-pass filter at 800 Hz.

EEG data were digitally bandpass filtered offline, with a Hann function filter at 0.5 Hz and 250 Hz, with a tapering window at 10%. No notch filter was applied.

### 2.4 Data preparation and analyses

The data were prepared as follows. Event related potential (ERP) averages were extracted from epochs of -500 to 6000 milliseconds relative to the stimulus onset, then baseline-corrected using the -500 millisecond interval before the stimulus onset. Each epoch was extracted at exactly one of the trigger events where a single image stimulus provoked phase-locked averaging (Fig 1B). Frequency spectra, in the frequency range from 0.5 Hz to 250 Hz, were computed by Fourier transformation for ERP averages at all 64 electrodes in the CURRY 7.4 Neuroscan. Spectral power values were computed in intervals covering the stimulation flicker rate frequencies used (8 Hz and 36 Hz). The alpha power values (corresponding to 8 Hz stimulation) were measured in the interval 6–10 Hz, and the gamma power values (corresponding to the 36 Hz stimulation) were measured in the 30–40 Hz interval. We use the maximal power observed within the intervals as our power measure. Since the power distribution is very narrow (i.e., not flat) and the maximal power is observed very close to the frequency of the stimulation (8 or 36 Hz), the width of the band should be inconsequential for most reasonable ranges of possible bandwidths. The spectral values were multiplied by their frequency to correct for the inverse frequency (1/*f*) characteristic of the typical frequency spectrum. All references to “power” or “log power” in this paper refer to the corrected power, or the natural logarithm of the corrected power.

Before analyzing the data, epochs were visually inspected for muscle artifacts, bad blocks, eye movements, EKG artifacts, and other detectable artifacts, which were then rejected using covariance methods. Data were also rejected if eye-movement artifacts or electrode drifts were visible in the data plots. In the cases where we rejected epochs, we rejected less than five and, and we rejected less than 100 epochs in total out of the 1600 person-epochs.

The variables used to replicate the baseline analysis of the present article can be found in S1 Data.

### 2.5 Artifacts

Initially, the data were visually inspected for obvious anomalies (such as an absence of a signal), which was not found in any of the measurements. The data was then corrected offline for artifacts, eye movements, and EKG artifacts using the covariance method incorporated in the software CURRY 7.4 Neuroscan. Artifact-affected intervals associated with eye-related movements were identified using thresholds established using the vertical eye electrodes (VEO), with the lower and upper thresholds set at -200 to 200 μV respectively. Voltages outside of this interval were defined as indicative of eye-related movements, and measurements in all channels in the time range -200 ms to 500 ms were then defined as potentially affected by artifacts. In a similar manner, we defined EKG-related artifact-affected intervals in the time range -200 ms to 500 ms, surrounding a QRS complex detection. The covariance method used to correct data in artifact-affected intervals involves covariance analyses between the artifact channel and each EEG channel, wherein linear transmission coefficients are computed, and, based on these, a proportion of the voltage is subtracted from each data point in the artifact interval.

### 2.6 Cognitive assessment of participants from the Metropolit 1953 Danish male birth cohort (Metropolit)

Participants (i.e., subject group 1) had their intelligence assessed using the IST2000-R test [45], with speed of processing tested using the trail-making test, and the symbol-digit modalities test (SDMT) [46;47]. Processing speed is a measure of visual attention, task switching, and visual scanning. The participants’ global neurocognitive functions were assessed with the mini-mental state examination (MMSE), and Addenbrookes cognitive examination (ACE). These scores were used to exclude and account for potential signs of dementia.

### 2.7 Statistics

Averaging, Fourier transformation, and power detection were performed with the CURRY 7.4 Neuroscan.

The main statistical analyses were performed using linear regression models to investigate the relationship between cognitive function and the SSVEP-PR. The linear regression model enabled us to estimate partial correlations between an outcome variable (i.e. SSVEP-PR or an intelligence test score) and one or more explanatory variables (i.e. age group, SSVEP-PR, and other variables). It should be noted that the linear regression model could be specified with both continuous variables as well as factor variables indicating groups (i.e., binary dummy variables). Thus, both continuous as well as group comparisons could be modeled. In order to test the statistical significance of the parameters used in the model, we performed two-sided *t*-tests, operating everywhere with a significance level of 5%. Coefficients with *p*-values of between 5% and 10% were denoted borderline significant. Confidence limits for proportions were calculated with Wald's method. We use heteroscedasticity-robust standard errors in all of the linear regression models in this paper, except when there are multiple observations per individual, in which case we use cluster-robust standard errors, clustered at the subject level. Since *t*-statistics, and thus *p*-values, are derived from the coefficient estimates and standard errors, we avoided redundancy by reporting coefficient estimates and standard errors in tables, while indicating the level of statistical significance using “***” to denote *p* < 0.01, “**” for *p* < 0.05, and “*” for *p* < 0.1. The adjusted *R*^2^ values were also reported in tabular form. All statistical analyses were performed in *SAS 9*.*4*.

We selected four main surface electrode areas of interest: the occipital region, the parietal region, the temporal region, and the frontal region.

**Fig 2:**
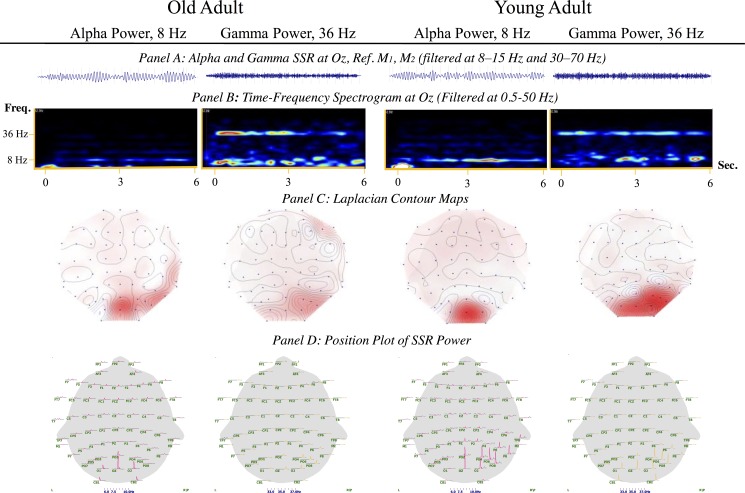
Illustration of the electrophysiological response (steady-state evoked potential) when stimulating with 8 Hz and 36 Hz in, respectively, a young and an old adult from our sample. Panel A) shows the amplitude at the Oz electrode, in the time domain filtered at the alpha range (8–12 Hz), and in the gamma range (30–70 Hz), with reference to M1 and M2. Panel B) shows time frequency spectrograms for the occipital electrodes (Oz) with data filtered at 0.5–250 Hz. The resolutions used are 126 ms in the alpha range, and 256 ms in the gamma range. The max frequency shown is 62.5 Hz. Note that stimulation with an 8 Hz flicker rate generated results suggestive of a sub-harmonic response. **Panel C)** shows 2D contour maps with a Laplacian transformation for 8 Hz (left) and 36 Hz (right). **Panel D)** shows 2D position plots of the spatial distribution filtered at 0.5–250 Hz, shown in the range of 6–10 Hz and 33–37 Hz. Clear peaks at 8 Hz and 36 Hz are seen for both age groups, with an indication of a greater response in the young adults.

The study consisted of two main statistical analyses. The first focused on the combined sample of young and old adults (i.e. cohorts 1 and 2 combined), and established the effects on power of the complex stimulation procedure. In this part of the analysis, we performed nonparametric Wilcoxon two-sample tests of the mean differences of the SSVEP-PRs for the young versus old subjects (data shown Fig 2). Furthermore, the SSVEP-PR data of the two age-groups were investigated using linear regression analyses, including factor variables inclusive of flicker-rate, the scalp region, and interaction terms.

Letting subscripts *i*, *r*, and *f*, denote the individual, scalp region, and stimulation frequency respectively, the general version of the linear regression model used in the first analysis to investigate the relationship between SSVEP-PR, age, and frequency is given by:

$$y_{i,r,f}=\beta_{0}+\beta_{1}x_{1,i,f}+\beta_{2}x_{2,i,f}+x_{3,i,r,f}^{\prime }\beta_{3}+x_{1,i,f}x_{3,i,r,f}^{\prime }\beta_{4}+\varepsilon_{i},$$

where *y*_*i*,*r*,*f*_ is the log power response in the scalp region *r* of individual *i* at frequency *f*, *x*_1,*i*,*r*,*f*_ is a dummy variable indicating if individual *i* is in the aged male group, *x*_2,*i*,*r*,*f*_ is a dummy variable indicating if the observation related to the high flicker-rate, ***x***_*3*,*i*,*r*_ is a vector of dummy variables indicating if the observation is related to the occipital, the parietal, the temporal, or the frontal scalp region, and *ε*_*i*_ is an error term that is clustered at the individual level. To assess the robustness of the results, we incorporated a varying number of explanatory variables.

The second part of the analysis focused on the older adults exclusively and investigated the association between SSVEP-PR and cognitive function using linear regression models.

Using the same terminology as above, the general version of the linear regression models used in the second analysis, that investigates the relationship between cognitive function and SSVEP-PR is given by:

$$y_{i}=\beta_{0}+\beta_{1}(x_{2,i}–x_{1,i})+\beta_{2}x_{1,i}+C_{3,i}^{\prime }\beta_{3}+\varepsilon_{i},$$

where *y*_*i*_ is a measure of cognitive function (an intelligence test score or a speed of processing test score), *x*_1,*i*_ is the ratio of the average power responses in the visual region to the average power response in a reference region, measured in the alpha range, i.e., based on the power response to the 8 Hz flicker rate (corresponding to *R*_*α*,*V*_ defined below), and *x*_2,*i*_ is the analogous measure in the gamma range, i.e. based on the power response to the 36 Hz flicker rate, (corresponding to *P*_*γ*,*V*_ defined below). The term ***C***_3_ is a vector of control variables, including a measure of global cognition, as well as measures of processing speed. We generally include the level, *x*_1,_ alongside the difference, *x*_*2*_ –*x*_*1*,_ which is our main variable of interest (corresponding to Δ*R*_*V*_ defined below), to account for the fact that the difference is likely to be correlated with the level, which may in turn be independently correlated with cognitive function.

### 2.8 Definitions

All power measures used in our statistical analyses are based on frequency–corrected power measures, i.e.,

$$P_{c}=P_{m}f,$$

where *P*_*c*_ is frequency-corrected power, *P*_*m*_ is measured power averaged over the electrodes, both of which were measured in squared microvolts, i.e., (*μV*)^2^, and *f* is the corresponding frequency.

We calculated the power responses as averages over certain scalp regions. The scalp regions of interest, investigated in our initial analyses, were defined by the following groups of electrodes:

Region of Interest:

*Visual-Area Electrodes (V)*: Oz, O1, O2, PO3, PO4, PO5, PO6, PO7, PO8

Four Main Regions:

*Occipital Electrodes (O)*: Oz, O1, and O2

*Parietal Electrodes (P)*: Pz, P1, P2, P3, P4, P5, P6, P7, and P8

*Temporal Electrodes (T)*: T7, T8, TP7, and TP8

*Frontal Electrodes (F)*: Fz, F1, F2, F3, F4, F5, F6, F7, and F8

Reference Area:

*Reference Electrodes (R)*: All main electrodes except those listed in *F* and *V*

Due to the prominence of the visual system in relation to the visual nature of the stimulations used, we anticipated that the relative visual-area response to (the stimulations used) would present a stronger correlation with intelligence than that of the other areas in the brain. Thus, in the main analysis, as will be explained further below, our variable of interest is the power response in the visual-area electrodes normalized by the power response in the reference electrodes. Furthermore, in some analyses, we also use the power response in the frontal electrodes, also here normalized by the power response in the reference electrodes.

The alpha-band relative visual-area power (denoted *R*_*α*,*V*_), and the gamma-band relative visual-area power (*R*_*γ*,*V*_) for each individual was defined by:

$$R_{\gamma ,V}=P_{\gamma ,V}/P_{\gamma ,R}$$

$$R_{\alpha ,V}=P_{\alpha ,V}/P_{\alpha ,R},$$

where *P*_*γ*,*V*_ is the average frequency-corrected power response to the 36 Hz stimulation at the visual-area electrodes, measured in the 30–40 Hz interval, *P*_*γ*,*R*_ is the average frequency-corrected power response to the 36 Hz stimulation at the reference electrodes, measured in the 30–40 Hz interval, *P*_*α*,*V*_ is the average frequency-corrected power response to the 8 Hz stimulation in the visual-area electrodes, measured in the 6–10 Hz interval, and *P*_*α*,*R*_ is the average frequency-corrected power response to the 8 Hz stimulation at the reference electrodes, measured in the 6–10 Hz interval.

For each individual, we defined the alpha-to-gamma difference in relative visual-area power. We denote this difference Δ*R* and define it formally by:

$$∆R_{V}=R_{\gamma ,V}-R_{\alpha ,V}.$$

Δ*R*_*V*_ is our main variable of interest. In a completely analogous way, we also defined the alpha-to-gamma difference in relative frontal power, which is denoted by Δ*R*_*F*_.

Furthermore, we used the following groups of neurocognitive tests (and subtests in parentheses):

*Intelligence*: IST2000-R (total, sentence completion, analogies, and numeric skills)

*Speed of Processing*: Trail-Making (A and B), and SDMT

*Global Cognition*: ACE and MMSE

To make the trail-making measures more intuitive, such that higher values represent faster task-completion, the signs of the trail-making variables were changed (i.e. the values of the Trail-Making A and B scores were multiplied by -1).

### 2.9 Ethics

The procedures are in accordance with the Declaration of Helsinki and the study was approved by the local ethical committee (De Videnskabsetiske Komiteer for Region Hovedstaden) and registered by the Danish Data Protection Agency. All participants provided written informed consent prior to study commencement.

## 3 Results

In this section, we present our findings. Before turning to the main findings in section 3.2, we first present in section 3.1 some initial findings on the age-dependence of the level of power across main regions in the brain (section 3.1.1) as well as of our measure of interest (section 3.1.2).

To put the results in context, we note that for all five regions, the alpha frequency response is the highest. This is also the case for the young, except for the visual area, where the response is highest for the gamma frequency. However, in the latter case, the difference is very small (i.e., less than 4% smaller at the alpha frequency).

### 3.1 Age-dependence

#### 3.1.1 Main regions

To assess the general age differences in complex image processing, we compared the SSVEP power across the four main regions of a group of older adult men to a group of young volunteers.

Table 1 presents the results of linear regression models examining the SSVEP-PR in terms of age, frequency, scalp region, and gender, as well as gender interacted with region. We found a significantly lower SSVEP-PR in aged adults compared to younger adults (columns 1–4). Furthermore, the SSVEP-PR was significantly lower in response to stimulation with a higher flicker rate (columns 1–4), meaning that gamma power is generally lower than alpha power. We also found that SSVEP-PRs were lower in the parietal, temporal, and frontal regions compared to the occipital region, which is consistent with the visual nature of the stimulation (columns 2–4). Interestingly, these differences were diminished among older adults (as shown by the estimated coefficients of the interaction terms used in our regression models), indicating that the power response was more evenly distributed over the scalp in aged participants (columns 3–4).

**Table 1:** The association between power responses and age group, flicker-rate, as well as scalp regions.

Model	1	2	3	4
Old Adults *(Ref*. *Young)*	-0.53*** (0.21)	-0.53*** (0.21)	-1.12*** (0.41)	-1.33*** (0.43)
High Flicker Rate *(Ref*. *Low Flicker-rate)*	-0.56*** (0.11)	-0.56*** (0.11)	-0.56*** (0.11)	-0.56*** (0.11)
Parietal		-0.28*** (0.08)	-0.58*** (0.22)	-0.58*** (0.22)
Temporal		-1.22*** (0.12)	-2.00*** (0.41)	-2.00*** (0.41)
Frontal		-0.94*** (0.12)	-1.77*** (0.42)	-1.77*** (0.42)
Occipital		Ref.	Ref.	Ref.
Old Adults × Parietal			0.36 (0.23)	0.36 (0.23)
Old Adults × Temporal			0.93**(0.43)	0.93**(0.43)
Old Adults × Frontal			0.99**(0.43)	0.99**(0.43)
Old Adults × Occipital			Ref.	Ref.
Male *(Ref*. *Female)*				-0.43 (0.35)
Number of Individuals	64	64	64	64

*** *p* < 0.01 and

** *p* < 0.05.

Using linear regression we regressed the log power response on age group, flicker-rate, scalp regions, and interaction terms between age group and scalp region. The model includes a constant term that is omitted from the table. Standard errors clustered at the subject level are shown in parentheses.

Fig 2 depicts an illustration of the electrophysiological response (steady-state evoked potential) following stimulation at either 8 or 36 Hz in young and old adults respectively. These data show time-frequency spectrograms for the occipital electrodes, with position plots; S1 Fig depicts an illustration of the steady-state evoked response and time-frequency spectrograms for the midline electrodes (Oz, POz, Pz, CPz, Cz, FCz, Fz).

Our findings of a more even spatial distribution of power in aged adults supports using a *relative* power measure in the main analysis below.

#### 3.1.2 Measure of interest (ΔR_V_)

In this section, we investigate how relative visual-area power in the alpha and gamma bands, as well as our measure of interest (the alpha-to-gamma *difference* in relative visual-area power) is dependent on age. We include gender as a control variable in all specifications, and show the results with and without controlling for the number of years of education.

Table 2 establish using linear regression that the alpha frequency response did not differ significantly between the young versus the old cohort, regardless of whether we control for the number of years of education.

**Table 2:** Correlation between the difference in relative occipital power and age.

	*R*_*α*,*V*_		*R*_*γ*,*V*_		*ΔR*_*V*_	
	1	2	3	4	5	6
Old Adults *(Ref. Young)*	0.35 (0.65)	2.24 (1.59)	-2.87*(1.44)	-2.43 (1.67)	-3.22**(1.21)	-4.67**(1.88)
Gender *(Ref. Female)*	-1.30 (0.81)	-1.38*(0.80)	2.90**(1.45)	2.88*(1.44)	4.20***(1.30)	4.26***(1.35)
Years of Education		0.36 (0.22)		0.08 (0.10)		-0.27 (0.21)
Adjusted *R*^2^	-0.02	0. 05	0.13	0.13	0.06	0.09
Number of Individuals	64	64	64	64	64	64

*** *p* < 0.01

** *p* < 0.05, and

* *p* < 0.1.

Correlation between the difference in relative occipital power and age. Using linear regression, we regressed the relative SSVEP-PR in the alpha and gamma ranges (i.e., *R*_*α*,*V*_ and *R*_*γ*,*V*_), and the alpha-to-gamma difference in relative visual-area power (i.e., Δ*R*_*V*_), on dummy variables indicating age group and gender, as well as a variable measuring the years of education. The model includes a constant term that is omitted from the table. Standard errors clustered at the subject level are shown in parentheses. The table establishes that Δ*R*_*V*_ is associated with age. Furthermore, the table establishes that the association between Δ*R*_*V*_ and age is robust to controlling for years of education, unlike the level of power in the alpha and gamma ranges. These findings indicate that Δ*R*_*V*_ may be more important than the level of either response in relation to age and therefore also cognitive decline.

Furthermore, while there was a significant negative correlation between age and the gamma frequency response in the basic specification where we only control for gender, this relation was not robustly significant when also controlling for the number of years of education. This indicates that the level of the gamma response might not capture neurophysiological changes over the life-course, but rather differences in socioeconomic factors between young and old.

Interestingly, our variable of interest, the difference in relative power, Δ*R*_*V*_, showed a pronounced and *robust* negative correlation with age, indicating that the relationship between the gamma and alpha responses may be important in relation to cognitive changes over the life course or even cognitive function in general (*p* = 0.01 in column 7) and (*p* = 0.02 in column 8). Δ*R*_*V*_ remains significantly correlated with the age group even when controlling for years of education. This indicates that Δ*R*_*V*_ might capture neurophysiological changes that are not related to socioeconomic factors, meaning that our variable of interest could potentially be used as an indicator of inherent neurophysiological characteristics such as cognitive performance, as will be corroborated below. The coefficient for Δ*R*_*V*_ in Table 2 of -3.22 implies that Δ*R*_*V*_ falls on average more than one standard deviation from young to old adults (c.f. the standard deviation in S1 Table).

### 3.2 Δ*R*_*V*_ and cognitive performance

We analyze the association between intelligence test scores and our variable of interest, namely the alpha-to-gamma difference in relative visual-area power (i.e., Δ*R*_*V*_). This analysis is restricted to the sample of older adults, for whom basic characteristics together with cognitive performance data are known. S1 Table presents some descriptive statistics of the data.

Table 3 shows correlates between intelligence test scores and Δ*R*_*V*_. We show both the unconditional result as well as the results when controlling for a range of factors. In particular, we add an increasing set of control variables for global cognition (measured with ACE), processing speed (measured with Trail-Making A and SDMT), executive function (measured with Trail-Making B), and relative visual-area alpha power (i.e., *R*_*α*,*V*_). Furthermore, we also investigated an alternative measure, namely alpha-to-gamma difference in relative frontal power (i.e., Δ*R*_*F*_).

**Table 3:** Correlation between Δ*R*_*V*_ and intelligence test score.

	Overall Intelligence Score (Total Score)									
	1	2	3	4	5	6	7	8	9	10
Δ*R*_*V*_	-1.01***(0.26)		-1.02***(0.29)	-0.85***(0.27)	-0.80***(0.28)	-0.89***(0.25)	-0.85***(0.28)	-0.74***(0.26)	-1.92***(0.63)	-1.67***(0.59)
Δ*R*_*F*_		-0.81 (1.97)	0.27 (1.99)							
ACE				1.29***(0.27)				0.97***(0.26)		0.98***(0.26)
Trail-Making A^a^					0.46***(0.13)			0.12 (0.18)		0.16 (0.18)
Trail-Making B^a^						0.17***(0.06)		0.06 (0.06)		0.05 (0.06)
SDMT							0.52***(0.15)	0.18 (0.17)		0.17 (0.17)
*R*_*α*,*V*_									-1.03*(0.56)	-1.06*(0.53)
Semi-partial *R*^2^ of Δ*R*_*V*_	0.08		0.08	0.08	0.06	0.08	0.07	0.07	0.08	0.10
Semi-partial *R*^2^ of Δ*R*_*F*_		0.00								
Adjusted *R*^2^	0,07	-0,02	0.05	0.30	0.21	0.25	0.23	0.37	0.7	0.38
Number of Individuals	54	54	54	54	54	54	54	54	54	54

*** *p* < 0.01and

* *p* < 0.1.

^a^ Measured by test completion-time.

**Fig 3:**
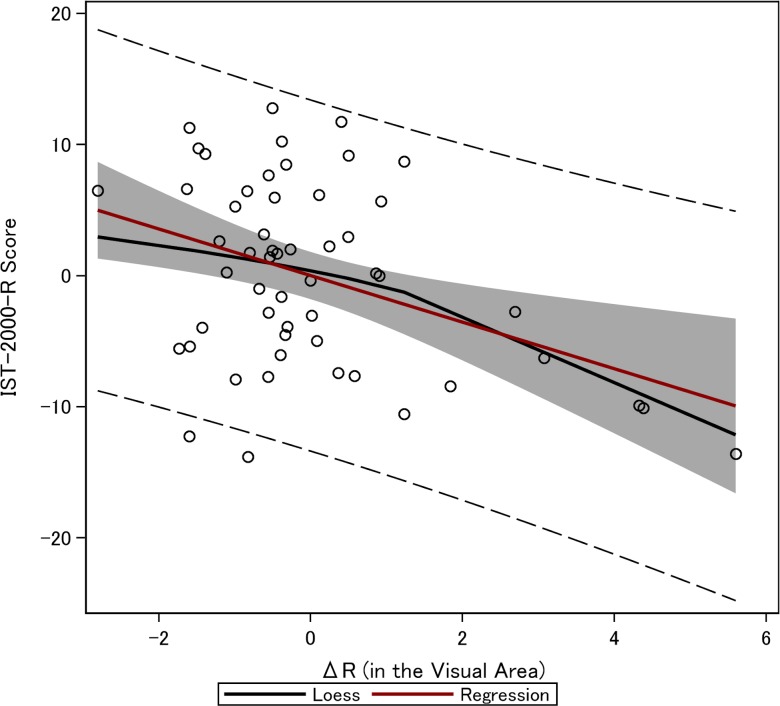
Added-variable plot of the partial correlation between intelligence (measured with IST-2000-R) and the alpha-to-gamma difference in relative visual-area power (Δ*R*_*V*_). The fit of the red line is generated by linear regression, estimated with OLS, when controlling for the individual alpha level (i.e., *R*_*α*,*V*_), global cognition (measured with ACE), and speed of processing (i.e., minus-Trail-Making A, minus-Trail-Making B, and SDMT) (see Table 3, Column 10). The black line is fitted with LOESS smoothing. The gray area represents the 95% confidence limit. The dotted lines represent the bounds of the 95% prediction limits.

Correlation between the alpha-to-gamma difference in relative visual-area power (Δ*R*_*V*_) and intelligence test score (IST-2000-R). Using linear regression we regressed intelligence scores for the total IST-2000-R on alpha-to-gamma difference in relative visual-area power (*ΔR*_*V*_). The model includes a constant term that is omitted from the table. Standard errors clustered at the subject level are shown in parentheses. Column 1 establishes the main result, that *ΔR*_*V*_ is significantly associated with the intelligence score, when controlling for alpha-to-gamma difference in relative frontal power (*ΔR*_*F*_) in column 3. Column 2 on the other hand show that alpha-to-gamma difference in relative frontal power (*ΔR*_*F*_) is not significantly associated with the intelligence score. Columns 5–10 show the robustness of the findings for *ΔR*_*V*_ when adding control variables for global cognition (ACE) and processing speed (minus-Trail-Making A, SDMT), executive function (minus-Trail-Making B) and while controlling for the individual alpha level, *R*_*α*,*V*_. The table establishes that the *ΔR*_*V*_ is robustly correlated with the total IST-2000-R score. An added-variable plot for the alpha-to-gamma difference in relative visual-area power and intelligence, corresponding to Column 10, is presented in Fig 3.

The alpha-to-gamma difference in relative visual-area power (Δ*R*_*V*_) was found to be negatively correlated with intelligence in all specifications (*p*<0.01 in all columns).

Interestingly, Δ*R*_*F*_ was not significantly correlated with intelligence (column 2). Further, when including both Δ*R*_*V*_ and Δ*R*_*F*_ in the same model, Δ*R*_*V*_ remained significant while Δ*R*_*F*_ remained insignificant (column 3).

When controlling for global cognition, executive function, and the speed of processing, Δ*R*_*V*_ remained significant (columns 4–8 and 10). In addition, when controlling for the relative visual-area alpha power, Δ*R*_*V*_ still remained significant (columns 9–10). It is worth noting that all cognition-related control variables are individually significantly positively associated with intelligence, as would be expected.

The coefficient of -1.67 for Δ*R*_*V*_ indicates that a one standard deviation higher Δ*R*_*V*_ is associated with an intelligence score lowered by 48% of a standard deviation (using the standard deviations reported in S1 Table). We therefore conclude that our data indicate a robust association between an individual’s intelligence and their alpha-to-gamma difference in relative visual-area power.

Furthermore, we calculated the area under the ROC sensitivity curve to investigate the accuracy of the main explanatory as a predictor of low IQ (defined as an IST-2000-R score lower than one standard deviation below the mean). We include *R*_*α*,*V*_ as a predictor (otherwise, the sensitivity would be lower). The raw sensitivity obtained without the use of any cognition-related control variable was 72% (*p* = 0.03), see S2 Fig Adding years of education, the sensitivity rises to 88% (*p*<0.0001).

To further investigate the results related to intelligence, we examined the sub-components of the IST-2000-R test, as shown in Table 4. The sub-component of IST-2000-R test are the “sentence completion” score, the “analogy” score, and the “numeric” score. The table establish that Δ*R*_*V*_ is significantly correlated with the “analogy” and the “numerical” score (columns 1–2). In contrast, the "sentence completion" portion of the verbal intelligence test was not significantly correlated with SSVEP-PR. We therefore conclude that the association between an individual’s intelligence and Δ*R*_*V*_ is mainly related to the “numeric” and the “analogy” scores in the IST-2000-R intelligence test.

**Table 4:** Correlation between Δ*R*_*V*_ for the three main parts of the intelligence test score.

	Analogy Score	Numeric Score	Sentence Completion Score
	1	2	3
*ΔR_V_*	-0.25***(0.08)	-0.45**(0.18)	-0.04 (0.08)
ACE	0.27**(0.12)	0.44***(0.14)	0.26***(0.09)
Trail-Making A^a^	-0.02 (0.07)	0.05 (0.10)	0.09 (0.06)
Trail-Making B^a^	0.03* (0.02)	0.03 (0.04)	-0.01 (0.02)
SDMT	0.09* (0.05)	0.10 (0.12)	-0.02 (0.05)
Semi-partial *R*^2^ of *ΔR_V_*	0.06	0.07	0.00
Adjusted *R*^2^	0.30	0.28	0.11
Number of Individuals	54	54	54

*** *p* < 0.01

** *p* < 0.05, and

* *p* < 0.1

^a^ Measured by test completion-time.

Correlation between alpha-to-gamma difference in relative visual-area power (Δ*R*_*V*_) for the three main parts (i.e., sentence, analogies, and numeric) of the intelligence test score (IST-2000-R). Using linear regression we regressed the three components of the IST-2000-R test on the Δ*R*_*V*_ while controlling for global cognition (ACE), processing speed (minus-Trail-Making A, SDMT) and executive function (minus-Trail-Making B). The model includes a constant term that was omitted from the table. Standard errors clustered at the subject level are shown in parentheses. The table establishes that the correlation is mainly attributed to the analogy and numbers part of the test, suggesting that the correlation between SSVEP-PR and intelligence is mainly related to the more logically demanding parts of the test.

In addition, S2 Table establishes that the results are robust to including a further extended set of control variables. In particular, we control for both shorter-run and longer-run memory test scores, and the number of years of education, in addition to the baseline control variables mentioned above. The correlation between intelligence and Δ*R*_*V*_ is remarkably robust and remain highly significant in the presence of all or our control variables.

Fig 3 shows the added variable plot corresponding to column 4 of S2 Table, which is the most comprehensive specification.

Moreover, S3 Table establishes that other brain areas (i.e., frontal, temporal, and parietal regions) do not yield significant correlations. In particular, when including measures that are analogous to our main variable of interest, but based on other brain regions, our main variable of interest is the only one that is significant.

Overall, we found that the difference in relative occipital power difference was highly significantly and robustly related to overall intelligence. Furthermore, it was most clearly related to a reduction in the more logically demanding aspects of the IQ-test. In addition, we found that the SSVEP-PR contains information that appears not to depend on global cognition, processing speed, executive function, memory, or education.

## 4 Discussion

This study presented two main findings. First, we found that alpha-to-gamma difference in relative visual-area power following visual stimulation is negatively correlated with age. Second, we found that the same measure is also robustly negatively correlated with intelligence, based upon data obtained by examining the Metropolit 1953 Danish Male Birth Cohort (Metropolit). These data indicate the potential for SSVEP-PR measurements to be used clinically to provide a non-subjective assessment of cognitive status in older age; further studies are now needed to develop this finding. In particular, we anticipate that further investigations of cognitive development throughout life with reference to power measurements in the visual area could be fruitful. Furthermore, it might be useful to investigate any potential confounding effects contributed by GABA concentration, the thickness of the parieto-occipital lobe, and brain-region volume.

We here discuss how our findings relate to two main types of factors that may be related to changes in cognitive function and EEG measurements. Firstly, socioeconomic factors may change over the life-course of individuals, or between cohorts, and these changes may ultimately be related to EEG measurements and intelligence, independently of age. Secondly, various neurophysiological phenomena not related to socioeconomic factors may explain changes in cognitive function or visually evoked EEG measurements over the life-course: (i) changes in neural density and connectivity, (ii) age-related decreases in the GABA-concentration, or (iii) potential changes in the retina of the eye which could affect the flicker-sensitive magnocellular pathways.

Socioeconomic factors may affect EEG measurements and may be bi-directionally causally related to intelligence. In particular, previous research has shown that individuals with different levels of measurement-related stress or kinds of socioeconomic background present different EEG characteristics [48–50]. Furthermore, these factors may themselves affect, or be affected by, intelligence test scores. We attempted to disentangle the correlation between our EEG measures and age, as well as the correlation between our main EEG measure and intelligence by controlling for the number of years of education completed by our study subjects. As mentioned above, we found that when controlling for years of education in the correlation between EEG measures and age, the otherwise significant correlation between gamma power and age was no longer significant, whereas our measure of interest (Δ*R*_*V*_) remained significantly correlated with age. In light of the research mentioned just above, we interpret this robustness of the association between our measure of interest and age as not being directly attributable to changes in socioeconomic factors over the life-course or across cohorts. In contrast, the correlation between gamma power and age found in this and other studies may simply reflect variations in socioeconomic status, consistent with an increase in average levels of education, such as that experienced by the Danish population over the past 40 years.

However, years of education may not capture all relevant socioeconomic aspects. For example, as described in [27], normal aging is associated with a slow decline in, e.g., the filter distracting internal and external stimuli. Clearly, the level of education may not capture all changes in this ability over the life-course or across cohorts. For example, it is possible that the ability to filter unnecessary stimuli is developing through the working years, and differentially so in individuals with different occupations. Therefore, although we find it promising that our main finding is robust to controlling for one important socioeconomic factor (education), we are aware that other secular changes in society may account for the significant difference in our main variable across the two cohorts. We therefore suggest that future studies of EEG in an age-related context attempt to control as much as possible for such societal changes.

Neurophysiological phenomena not related to socioeconomic elements may also act as confounding factors. For example, it is well known that while the gross morphological structure (e.g., size) of the brain changes relatively little between childhood and mid-adulthood, dramatic changes in neural density and connectivity are known to occur over this time frame [51–53], as well as in patients with mild cognitive impairment [33;54–59]. Therefore, one might hypothesize that the correlation between gamma band activity and age could be attributed to morphological changes. However, in one study investigating the associations between electrophysiological responses, MRI volumetric parameters, and age, Gaetz et al. found a negative correlation between gamma band activity and age that could not be attributed to age-related structural change [28].

It has been suggested that additional neural activity could also represent over-recruitment, which is detrimental to task performance [60]. Likewise, additional neural activity could also represent greater effort required to perform a task, or the non-beneficial recruitment of additional neural units [61;62]. Previous studies have also shown that there are differences in SSVEP measurements during memory activation in different age groups [3;30;63]. For example, it has been shown that younger adults demonstrated greater SSVEP amplitude and latency reduction during tasks involving working-memory than older adults. These data were interpreted to indicate an age-related decrease in neural processes due to reduced cortical activation in older adults.

Alpha oscillations are hypothesized to have a role in both bottom-up visual processing and perception, as well as attention selection and the inhibition of irrelevant information [64]. In this context, better oscillatory synchrony and phase alignment may reflect a greater effort in attentional selection. In addition, Alzheimer’s disease patients have increased power in the delta and theta bands, and decreased power in the alpha and beta bands [4–6;24–25].

In addition, a study on gratings-induced gamma frequency demonstrated a positive correlation with resting endogenous GABA concentration [65;66], something which also correlates negatively with normal aging [67;68]. Consequently, the (non-robust) indication of an age-related decrease in gamma frequency observed in our study may be related to age-related decrease in GABA concentration; alternatively, both gamma-band frequencies and GABA concentration may be sensitive to additional and related processes in the aging brain.

A correlation between intelligence and Δ*R*_*V*_ is consistent with a study using both MEG and MRS in humans, which demonstrated that orientation discrimination performance was predicted by both occipital GABA concentration and gamma-band frequency within the same area, thereby suggesting that variability of GABAergic inhibition can provoke variability in human performance [69].

Finally, potential changes in the retina of the eye may affect the flicker-sensitive magnocellular pathways and thereby the EEG measurements. Achromatic and low spatial contrast stimuli, such as the vase-face images, presented with a high frequency of 36 Hz, as used in our study design, should theoretically activate the magnocellular (MC) pathways. Because patients with Alzheimer’s disease have a predilection for the MC pathway, we suggest that our use of complex images at a varying flicker rate could be a useful tool to assess MC pathway activity in patients with a possible diagnosis of Alzheimer’s disease [70]. Overall, neocortical gamma EEG activity is crucial for sensory perception and cognition, and in Alzheimer’s disease its disruption leads to impaired information processing.

This paper contributes to the literature on cognitive performance and EEG measures. A growing body of evidence suggests that activity in the gamma-band may be a key neural signature of information processing in the mammalian brain, which would be consistent with our findings. For example, gamma-band responses have been associated with higher-order cognitive functions, such as perception [35–37] and memory [38]. Gamma oscillations have also been proposed as a fundamental mechanism for cortical computation and long-range communication between brain areas [39]. However, relatively little is known about gamma responses over the lifetime of subjects [28–31]. Findings of power differences between young and old adults shed light on correlations between age and gamma band activity. Our findings sheds light on a recent studies that reported a decrease in power in the gamma-band with age in a healthy adult population [32], and comparable studies based on animals [29;33]. In an attempt to control for the potential confounding effects of age, gender, and socio-economic differences, we investigated a homogenous cohort of older men, and derived correlates for measured SSVEP power with neurocognitive performance. Interestingly, when we investigated this uniform group of males born in the same year, and area, we found an inverse correlation between the alpha-to-gamma difference in relative visual-area power and the intelligence. This may indicate that subjects with a diminished cognitive performance may require greater relative visual-area effort to process even simple passive stimuli.

The finding of a negative association between our measure and intelligence indicates that brains which allocate relatively more effort in the visual area when increasing visual stimulus frequency are less intelligent. We interpret this as being consistent with a notion that more intelligent brains are not expending more effort when more complicated (high-frequency) input is processed, but that they are more efficient in processing that input. However, this interpretation rests on the assumption that passive observation of a flickering image at a higher flickering frequency is more cognitively demanding than observing an image that is flickering at a lower rate (this could for example be the case if it is more difficult for the brain to determine if the image is flickering at all at higher frequencies).

If the present results can be verified in future research, it might be interesting to study if cognitive development at the individual level could also be explained by similar SSVEP-PR measures. Furthermore, it would be interesting to investigate the robustness of our results with respect to alternative setups. For example, we referenced the signals to the mastoid electrodes out of concern for clinical relevance, but future research could investigate if this is a necessary condition for our findings, or if the results can also be established with a theoretically more satisfying technique, such as REST [71;72].

## 5 Conclusion

This study found that our novel non-task-based measure, Δ*R*_*V*_, is highly significantly and robustly correlated with intelligence. This finding was found to hold, even following corrections for processing speed, global cognition, executive function, memory, and education. Finally, ROC analyses showed that our EEG measures could predict lower cognitive function with a sensitivity of 72%. Furthermore, we report that Δ*R*_*V*_ was found to be age dependent.

We conclude that Δ*R*_*V*_ might prove useful in deriving non-subjective assessments of cognitive ability, and could potentially help identify discrete changes in otherwise normally functioning, aged adults.

## Supporting information

S1 Data This data file contains the variables required to replicate the baseline analysis. (ZIP)

S1 Fig Illustration of the electrophysiological response (steady-state evoked potential) to either 8 or 36 Hz stimuli in young versus old adults. Panel A) Amplitude at the central electrodes (Oz, POz, Pz, CPz, Cz, FCz, Fz) in the time domain filtered for the alpha range (8–12 Hz), and at the gamma range (30–70 Hz), with reference to M1 and M2. The time-frequency spectrogram is shown for the same electrodes, at a resolution of 256 mms, and a maximal frequency of 15.6 Hz. Panel B) Amplitude at the central electrodes (Oz, POz, Pz, CPz, Cz, FCz, Fz) in the time domain filtered at the gamma range (30–70 Hz), with reference to M1 and M2. The time-frequency spectrogram is shown for the same electrode positions with a resolution of 126 ms, and a maximal frequency of 62.5 Hz.
(TIFF)

S2 Fig ROC sensitivity curve for as a predictor of low IQ. ROC sensitivity curve for the main explanatory variable (alpha-to-gamma difference in relative visual-area power), controlling for alpha-band relative visual-area power, as a predictor of low IQ (as defined by an IST-2000-R score lower than one standard deviation below the mean). The raw EEG-related sensitivity obtained (using Δ*R*_*V*_ and *R*_*α*,*V*_), without the use of any control variables, was 72% (*p* = 0.03). It rises to 88% when also including the number of years of education (*p*<0.0001).
(TIFF)

S1 Table Basic characteristics of the study population. (DOCX)

S2 Table Δ*R*_*V*_ and intelligence, controlling for education, memory, global cognition and speed of processing. Correlation between the alpha-to-gamma difference in relative visual-area power and the intelligence test score (IST-2000-R) while controlling for years of education, memory test scores, as well as the global cognition test score and the speed of processing test score. Using linear regression, we regressed intelligence scores for the total IST-2000-R score on the alpha-to-gamma difference in relative visual-area (Δ*R*_*V*_). The model includes a constant term that is omitted from the table. Standard errors clustered at the subject level are shown in parentheses. Column 1 establishes the main result, that alpha-to-gamma difference in relative visual-area power is significantly associated with the intelligence score, controlling for years of education. Column 2 establishes that Δ*R*_*V*_ is significantly associated with the intelligence score, controlling for memory measured with the paired associative learning test (PAL) by the number of errors during the learning and retention phase. Column 3 show that the findings are robust when including both the education and memory control variables. Column 4 show the robustness of the findings when including all the additional control variables in addition to all the baseline control variables. Columns 5–7 show the correlation between Δ*R*_*V*_ for the three main parts (i.e., sentence, analogies, and numeric) of the intelligence test score (IST-2000-R) when including all the control variables. The table establishes that our SSVEP-PR measure of interest is robustly correlated with the total IST-2000-R score conditional on a wide range of control variables.
(DOCX)

S3 TableIntelligence and power response measures for the four main regions of interest.The table shows the results of linear regression models of the test scores of intelligence and the visual evoked power responses for the four main regions of interest (i.e., $\Delta {\tilde{R}}_{V}$, $\Delta {\tilde{R}}_{P}$, $\Delta {\tilde{R}}_{T}$, $\Delta {\tilde{R}}_{F}$, please find the definitions of these variables below). Furthermore we subdivided $\Delta {\tilde{R}}_{V}$, into $\Delta {\tilde{R}}_{O}$ and $\Delta {\tilde{R}}_{Po}$ (please find the definitions of these variables below). Column 7–9 show a “horse race” regression with all four regions of interest included in the same model. The table establishes that the alpha-to-gamma difference in relative visual-area power remains significantly negatively correlated while the coefficients on the differences in the other brain regions are not significant. We define $\Delta {\tilde{R}}_{i}$ = *P*_*ie*_*/P*_*γ*,*j*_—*P*_*α*,*i*_*/P*_*α*,*j*_, where *i* is the region of interest, i.e., *V*, *O*, *P*, *T*, *F* (see Section 2.8 in the main article), or *PO*, which are the electrodes in *V*, except for those also in *O*, and *j* is the reference area consisting of the central electrodes: C5, C3, C1, Cz, C2, C4, C6, Cp5, Cp3, Cp1, Cpz, Cp2, Cp4, and Cp6.(DOCX)
