# Supplementary figures and images for: Visual steady state in relation to age and cognitive function
Source: PLoS One. 2017 Feb 28;12(2):e0171859. doi: 10.1371/journal.pone.0171859 (PMC5330460; doi:10.1371/journal.pone.0171859)

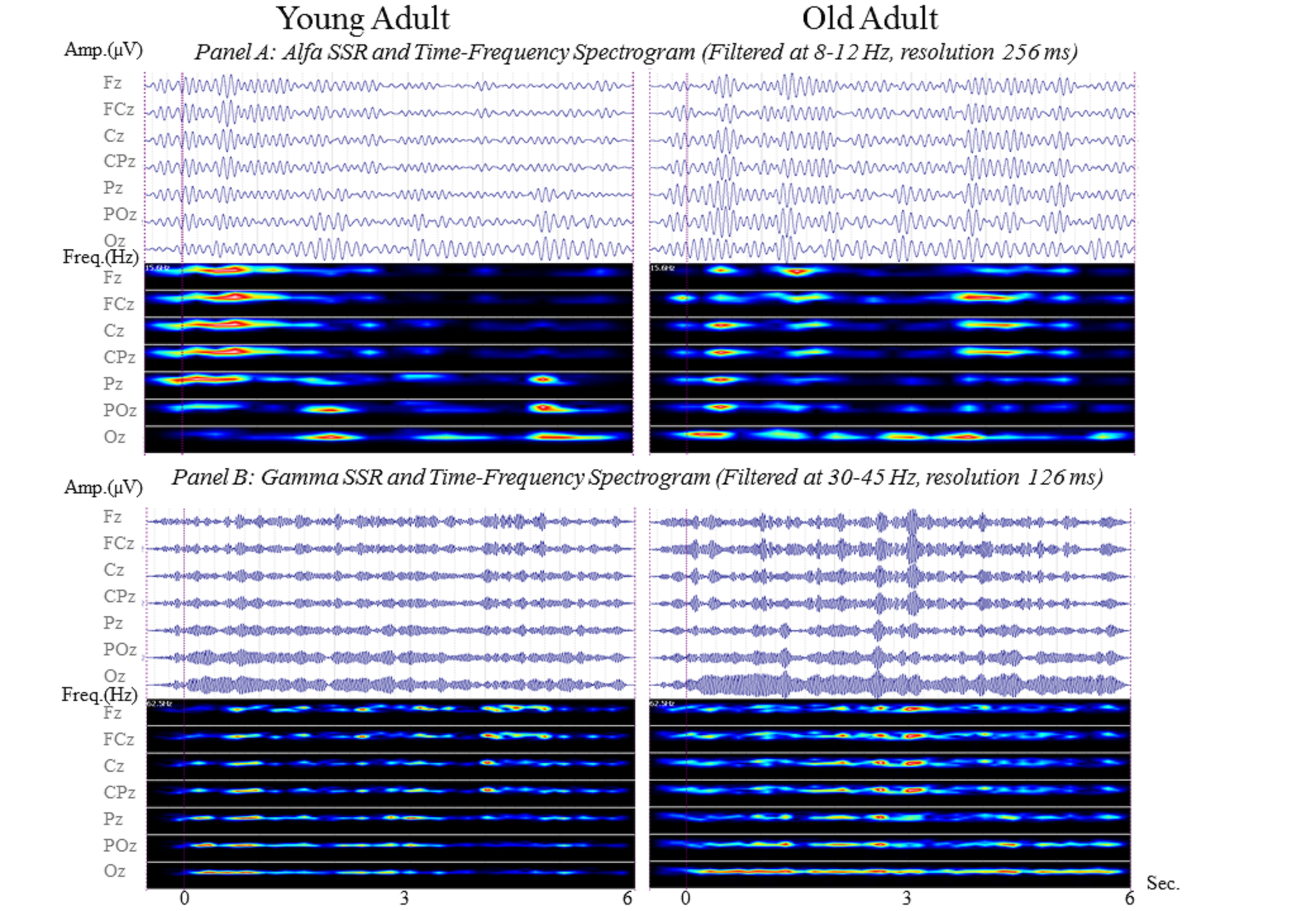

Supplement: S1 Fig — Panel A) Amplitude at the central electrodes (Oz, POz, Pz, CPz, Cz, FCz, Fz) in the time domain filtered for the alpha range (8–12 Hz), and at the gamma range (30–70 Hz), with reference to M1 and M2. The time-frequency spectrogram is shown for the same electrodes, at a resolution of 256 mms, and a maximal frequency of 15.6 Hz. Panel B) Amplitude at the central electrodes (Oz, POz, Pz, CPz, Cz, FCz, Fz) in the time domain filtered at the gamma range (30–70 Hz), with reference to M1 and M2. The time-frequency spectrogram is shown for the same electrode positions with a resolution of 126 ms, and a maximal frequency of 62.5 Hz. (TIFF) [file pone.0171859.s002.tiff]

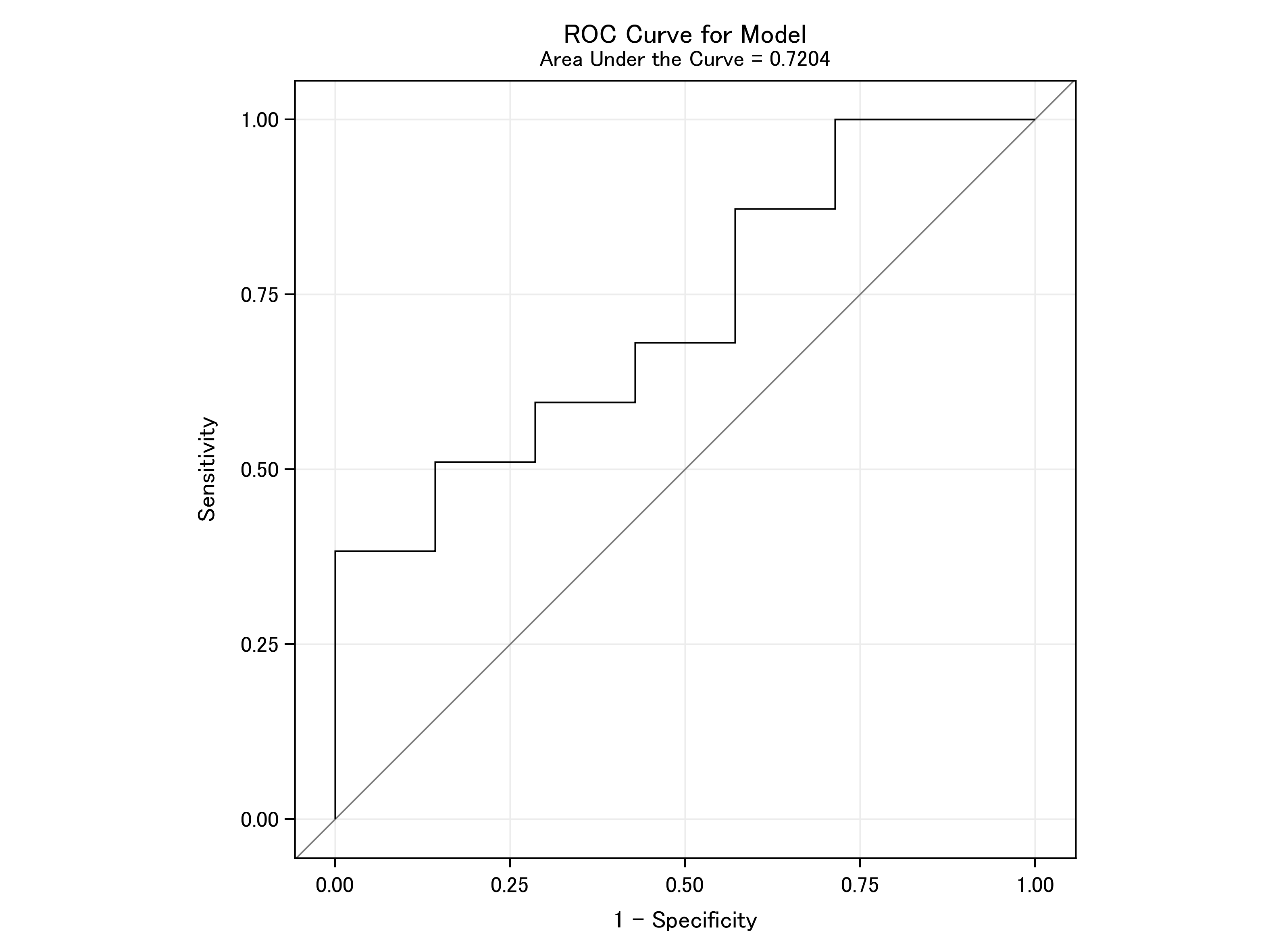

Supplement: S2 Fig — ROC sensitivity curve for the main explanatory variable (alpha-to-gamma difference in relative visual-area power), controlling for alpha-band relative visual-area power, as a predictor of low IQ (as defined by an IST-2000-R score lower than one standard deviation below the mean). The raw EEG-related sensitivity obtained (using ΔRV and Rα,V), without the use of any control variables, was 72% (p = 0.03). It rises to 88% when also including the number of years of education (p<0.0001). (TIFF) [file pone.0171859.s003.tiff]
