# Supplementary material for: Visual steady state in relation to age and cognitive function
Source: PLoS One. 2017 Feb 28;12(2):e0171859. doi: 10.1371/journal.pone.0171859 (PMC5330460; doi:10.1371/journal.pone.0171859)
Supplement: S1 Table — (DOCX) [file pone.0171859.s004.docx]

**S1 Table. Basic characteristics of the study population.**

|  | | Young Adults:  Cohort 1 | Old Adults:  Cohort 2 |
| --- | --- | --- | --- |
| Number of Subjects | | 10 | 54 |
| Number of Males (Percentages in Parentheses) | | 5  (50%) | 54  (100%) |
| Mean Age in Years (Range in Parentheses) | | 27.6  (25.0 to 34.0) | 62.01  (61.3 to 62.6) |
| Right Handed | | 80 % | 86 % |
| Electrophysiological measures Mean score (standard deviations in parentheses) | | | |
| Δ*R_V_* | | 0.99  (3.09) | -0.12  (3.10) |
| *R_α,V_* | | 2.86  (1.52) | 2.56  (3.20) |
| *R_γ,V_* | | 3.85  (2.85) | 2.43  (1.69) |
| Neurocognitive tests Mean score (standard deviations in parentheses) | | | |
| Short Cognitive Tests | Mini-Mental State Examination | - | 29.57 (0.69) |
|  | Addenbrooke’s Cognitive Examination | - | 94.69 (4.14) |
| Speed of  Processing | Trail-Making Test A | - | 32.70 (9.63) |
|  | Trail-Making Test B | - | 78.48 (27.81) |
|  | Symbol-Digit Modalities Test | - | 45.07 (8.69) |
| Intelligence | IST2000-R (total) | - | 34.05 (10.88) |
|  | IST2000-R (sentences) | - | 12.78 (3.10) |
|  | IST2000-R (analogues) | - | 10.89 (3.93) |
|  | IST2000-R (numbers) | - | 10.30 (6.08) |
