# Supplementary material for: Visual steady state in relation to age and cognitive function
Source: PLoS One. 2017 Feb 28;12(2):e0171859. doi: 10.1371/journal.pone.0171859 (PMC5330460; doi:10.1371/journal.pone.0171859)
Supplement: S2 Table — Correlation between the alpha-to-gamma difference in relative visual-area power and the intelligence test score (IST-2000-R) while controlling for years of education, memory test scores, as well as the global cognition test score and the speed of processing test score. Using linear regression, we regressed intelligence scores for the total IST-2000-R score on the alpha-to-gamma difference in relative visual-area (ΔRV). The model includes a constant term that is omitted from the table. Standard errors clustered at the subject level are shown in parentheses. Column 1 establishes the main result, that alpha-to-gamma difference in relative visual-area power is significantly associated with the intelligence score, controlling for years of education. Column 2 establishes that ΔRV is significantly associated with the intelligence score, controlling for memory measured with the paired associative learning test (PAL) by the number of errors during the learning and retention phase. Column 3 show that the findings are robust when including both the education and memory control variables. Column 4 show the robustness of the findings when including all the additional control variables in addition to all the baseline control variables. Columns 5–7 show the correlation between ΔRV for the three main parts (i.e., sentence, analogies, and numeric) of the intelligence test score (IST-2000-R) when including all the control variables. The table establishes that our SSVEP-PR measure of interest is robustly correlated with the total IST-2000-R score conditional on a wide range of control variables. (DOCX) [file pone.0171859.s005.docx]

**S2 Table. Δ*R_V_* and intelligence, controlling for education, memory, global cognition and speed of processing.**

|  | Overall Intelligence Score  (Total Score) | | | | Sentence Completion Score | Analogy Score | Numeric Score |
| --- | --- | --- | --- | --- | --- | --- | --- |
|  | **1** | **2** | **3** | **4** | **5** | **6** | **7** |
| Δ*R_V_* | **-0.52*****  (0.17) | **-0.82*****  (0.20) | **-0.52*****  (0.18) | **-0.42****  (0.18) | 0.04  (0.05) | **-0.15****  (0.06) | **-0.31*****  (0.12) |
| Years of Education | **2.54*****  (0.26) |  | **1.97*****  (0.28) | **1.74*****  (0.26) | **0.5*****  (0.08) | **0.41*****  (0.10) | **0.81*****  (0.17) |
| Memory, learning phase  (No. of Errors) |  | **-0.66*****  (0.13) | **-0.48*****  (0.14) | **-0.41*****  (0.11) | -0.06  (0.04) | **-0.09***  (0.05) | **-0.26*****  (0.07) |
| Memory, retention phase  (No. of Errors after 1 hour ) |  | 0.47  (0.41) | 0.51  (0.43) | **1.11*****  (0.35) | **0.24***  (0.13) | -0.10  (0.17) | **0.97*****  (0.23) |
| ACE |  |  |  | **0.89*****  (0.13) | **0.25*****  (0.05) | **0.20*****  (0.06) | **0.44*****  (0.07) |
| Trail-Making A^a^ |  |  |  | **0.22****  (0.08) | **0.11*****  (0.03) | 0.00  (0.03) | **0.12****  (0.05) |
| Trail-Making B^a^ |  |  |  | 0.00  (0.03) | **-0.02***  (0.01) | 0.01  (0.01) | 0.01  (0.03) |
| SDMT |  |  |  | 0.06  (0.08) | **-0.05***  (0.03) | **0.07****  (0.03) | 0.04  (0.07) |
| Semi-partial *R*^2^ of Δ*R_V_* | 0.02 | 0.05 | 0.02 | 0.02 | 0.00 | 0.02 | 0.03 |
| Number of Individuals | 54 | 54 | 54 | 54 | 54 | 54 | 54 |

*** *p*< 0.01, ** *p*< 0.05, and * *p*< 0.1 ^a^Measured by test completion-time.

**S2 Table legend:** Correlation between the alpha-to-gamma difference in relative visual-area power and the intelligence test score (IST-2000-R) while controlling for years of education, memory test scores, as well as the global cognition test score and the speed of processing test score. Using linear regression, we regressed intelligence scores for the total IST-2000-R score on the alpha-to-gamma difference in relative visual-area (Δ*R_V_*). The model includes a constant term that is omitted from the table. Standard errors clustered at the subject level are shown in parentheses. Column 1 establishes the main result, that alpha-to-gamma difference in relative visual-area power is significantly associated with the intelligence score, controlling for years of education. Column 2 establishes that Δ*R_V_* is significantly associated with the intelligence score, controlling for memory measured with the paired associative learning test (PAL) by the number of errors during the learning and retention phase. Column 3 show that the findings are robust when including both the education and memory control variables. Column 4 show the robustness of the findings when including all the additional control variables in addition to all the baseline control variables. Columns 5–7 show the correlation between Δ*R_V_* for the three main parts (i.e., sentence, analogies, and numeric) of the intelligence test score (IST-2000-R) when including all the control variables. The table establishes that our SSVEP-PR measure of interest is robustly correlated with the total IST-2000-R score conditional on a wide range of control variables.
