# Supplementary material for: Visual steady state in relation to age and cognitive function
Source: PLoS One. 2017 Feb 28;12(2):e0171859. doi: 10.1371/journal.pone.0171859 (PMC5330460; doi:10.1371/journal.pone.0171859)
Supplement: S3 Table — The table shows the results of linear regression models of the test scores of intelligence and the visual evoked power responses for the four main regions of interest (i.e., ΔR˜V, ΔR˜P, ΔR˜T, ΔR˜F, please find the definitions of these variables below). Furthermore we subdivided ΔR˜V, into ΔR˜O and ΔR˜Po (please find the definitions of these variables below). Column 7–9 show a “horse race” regression with all four regions of interest included in the same model. The table establishes that the alpha-to-gamma difference in relative visual-area power remains significantly negatively correlated while the coefficients on the differences in the other brain regions are not significant. We define ΔR˜i = Pie/Pγ,j—Pα,i/Pα,j, where i is the region of interest, i.e., V, O, P, T, F (see Section 2.8 in the main article), or PO, which are the electrodes in V, except for those also in O, and j is the reference area consisting of the central electrodes: C5, C3, C1, Cz, C2, C4, C6, Cp5, Cp3, Cp1, Cpz, Cp2, Cp4, and Cp6. (DOCX) [file pone.0171859.s006.docx]

**S3 Table:** Intelligence and power response measures for the four main regions of interest.

|  | Overall Intelligence Score  (Total Score) | | | | | | | | |
| --- | --- | --- | --- | --- | --- | --- | --- | --- | --- |
|  | **1** | **2** | **3** | **4** | **5** | **6** | **7** | **8** | **9** |
| Δ$\tilde{R}$*_O_* | **-0.38*****  (0.08) |  |  |  |  |  | **-0.39****  (0.15) |  |  |
| Δ$\tilde{R}$*_Po_* |  | **-0.53*****  (0.16) |  |  |  |  |  | **-0.73****  (0.28) |  |
| Δ$\tilde{R}$*_V_* |  |  | **-0.49*****  (0.12) |  |  |  |  |  | **-0.59****  (0.22) |
| Δ$\tilde{R}$*_P_* |  |  |  | -1.12  (1.22) |  |  | -0.22  (1.42) | 0.96  (1.58) | 0.44  (1.52) |
| Δ$\tilde{R}$*_T_* |  |  |  |  | 0.06  (0.65) |  | 0.21  (0.70) | -0.15  (0.73) | 0.03  (0.71) |
| Δ$\tilde{R}$*_F_* |  |  |  |  |  | -1.15  (1.87) | 0.50  (1.89) | 0.47  (1.95) | 0.53  (1.92) |
| Semi-partial *R*^2^ of Δ$\tilde{R}$*_O_* | 0.08 |  |  |  |  |  | 0.06 |  |  |
| Semi-partial *R*^2^ of Δ$\tilde{R}$*_Po_* |  | 0.08 |  |  |  |  |  | 0.07 |  |
| Semi-partial *R*^2^ of Δ$\tilde{R}$*_V_* |  |  | 0.09 |  |  |  |  |  | 0.07 |
| Semi-partial *R*^2^ of Δ$\tilde{R}$*_P_* |  |  |  | 0.02 |  |  |  |  |  |
| Semi-partial *R*^2^ of Δ$\tilde{R}$*_T_* |  |  |  |  | 0.00 |  |  |  |  |
| Semi-partial *R*^2^ of Δ$\tilde{R}$*_F_* |  |  |  |  |  | 0.01 |  |  |  |
| Adjusted *R*^2^ | 0.06 | 0.07 | 0.07 | 0.00 | -0.02 | -0.01 | 0.02 | 0.02 | 0.01 |
| Number of Individuals | 54 | 54 | 54 | 54 | 54 | 54 | 54 | 54 | 54 |

*** *p*< 0.01, ** *p*< 0.05, and * *p*< 0.1

**S3 Table legend:** The table shows the results of linear regression models of the test scores of intelligence and the visual evoked power responses for the four main regions of interest (i.e., Δ$\tilde{R}$*_V_* , Δ$\tilde{R}$*_P_* , Δ$\tilde{R}$*_T_* , Δ$\tilde{R}$*_F_* ). Furthermore vi subdivided Δ$\tilde{R}$*_V_* , into Δ$\tilde{R}$*_O_* and Δ$\tilde{R}$*_Po_*. Column 7–9 show a “horse race” regression with all four regions of interest included in the same model. The table establishes that the alpha-to-gamma difference in relative visual-area power remains significantly negatively correlated while the coefficients on the differences in the other brain regions are not significant. We define Δ$\tilde{R}$*_i_* = *P_ie_/P_γ,j_* - *P_α,i_/P_α,j_*, where *i* is the region of interest, i.e., *V*, *O*, *P*, *T*, *F* (see Section 2.8 in the main article), or *PO*, which are the electrodes in *V*, except for those also in *O*, and *j* is the reference area consisting of the central electrodes: C5, C3, C1, Cz, C2, C4, C6, Cp5, Cp3, Cp1, Cpz, Cp2, Cp4, and Cp6.
